# Nomograms for stratified prognosis prediction of gastric cancer by integrating programmed death ligand 1 and tumor-infiltrating immune cells including CD4+/CD8+ TILs and CD163+ TAMs

**DOI:** 10.3389/fonc.2025.1530054

**Published:** 2025-06-25

**Authors:** Xiumin Qi, Yixuan Guo, Yan Xiao, Xiang Pan, Fangming Chen, Xu Zhang

**Affiliations:** ^1^ Department of Pathology, Wuxi No.2 People’s Hospital, Jiangnan University Medical Center, Affiliated Wuxi Clinical College of Nantong University, Jiangnan University, Wuxi, Jiangsu, China; ^2^ Department of Radiology, Wuxi No.2 People’s Hospital, Jiangnan University Medical Center, Affiliated Wuxi Clinical College of Nantong University, Jiangnan University, Wuxi, Jiangsu, China; ^3^ School of Artificial Intelligence and Computer Science, Jiangnan University, Wuxi, Jiangsu, China; ^4^ Clinical Medical Research Center, Wuxi No.2 People’s Hospital, Jiangnan University Medical Center, Affiliated Wuxi Clinical College of Nantong University, Jiangnan University, Wuxi, Jiangsu, China

**Keywords:** gastric cancer, programmed death-ligand 1, tumor-infiltrating lymphocytes, tumor-associated macrophages, prognosis, nomogram

## Abstract

**Purpose:**

To develop nomograms for predicting disease-free survival (DFS) and overall survival (OS) of gastric cancer (GC) by integrating programmed death ligand 1 (PD-L1) and CD4+/CD8+ tumor-infiltrating lymphocytes (TILs) and CD163+ tumor-associated macrophages (TAMs).

**Materials and methods:**

Immunohistochemistry for PD-L1, CD4+/CD8+ TILs and CD163+TAMs was performed on 126 surgically-resected GC specimens between January 2016 and May 2018. Subsequently, the expression of PD-L1 and these tumor-infiltrating immune cells(TIICs), in combination with multiple clinicopathologic features, was used to formulate nomograms for predicting DFS or OS based on the results of multivariate Cox regression analysis. The performance of the nomograms for DFS or OS was verified in the 10-fold cross-validation of the study cohort and measured by Harrell’s concordance-index (C-index).

**Results:**

After multivariable Cox regression analyses, high PD-L1 expression (hazard ratio[HR]=2.17, 95% confidence interval [CI] 1.37–3.43), low CD8+ TILs density(HR=0.35, 95% CI 0.15–0.81), high CD163+ macrophages density (HR=1.84, 95% CI 1.17–2.89), TNM stage (stage III vs stage I+II, HR=1.37, 95% CI 1.06–2.23) and microsatellite instability-high(MSI-H) ( MSI-H VS microsatellite stability (MSS), HR=0.41, 95% CI 0.20–0.83) were found to be independent risk factors for DFS. Similarly, high PD-L1 expression (HR=2.64, 95% CI 1.61–4.34), high CD4+ TILs density (HR=1.98, 95% CI 1.21–3.24), low CD8+ TILs density (HR=0.23 95% CI 0.07–0.73), high CD163+ TAMs density (HR=2.31, 95% CI 1.43–3.74), MSI-H (MSI-H VS MSS, HR=0.26, 95% CI 0.12–0.60) and TNM stage (stage III vs stage I +II, HR=1.61, 95% CI 1.01–2.56) were independently associated with OS. These actors were then selected to establish nomograms for DFS and OS individually. The established nomogram for DFS yielded a corrected C-index of 0.679 by 10- fold cross-validation. Similarly, the established nomogram for OS yielded a corrected C-index of 0.755.These results suggest that PD-L1 and high density of CD4+ TILsas well as CD163+ TAMs are risk factors for poor prognosis in GC patients.On the contrary, MSI-H and high density of CD8+ TILsare associated with good prognosis in GC patients.

**Conclusions:**

The developed prognostic nomograms for GC integrating PD-L1 and CD4+/CD8+ TILs as well as CD163+TAMs offer a more personalized and precise prediction of DFS and OS for patients, which can help to improve prognostic stratification.

## Introduction

Gastric cancer (GC) is the fifth most common cancer and the third leading cause of cancer-related death (1–3). The combination of radical surgical resection (R0) plus postoperative chemotherapy is currently considered the primary method of GC treatment to achieve long-term survival, but survival outcomes vary (4–6). The tumor–node–metastasis (TNM) staging system and histologic classification are routinely used to predict prognosis (7, 8). However, considering the variations in clinical outcomes even in patients with similar disease TNM characteristics, risk-stratification tools for treatment decisions are still suboptimal (9, 10). Novel biomarkers are needed to improve the stratification of GC for accurately predicting patient prognosis (11–13).

The tumor microenvironment (TME) plays a crucial role in understanding the relationship between the immune system and the tumor (14–16). TILsand TAMs are essential components of the TME. There are multiple subtypes in TILs, such as CD4+ T cells, CD8+ T cells, and CD20+ B cells. TAMs can be divided into M1 subtype(CD68+ TAMs) and M2 subtype(CD163+ TAMs). Studies have revealed that the condition of CD4+/CD8+ TILs and CD163+ TAMs is a major hallmark of GC prognosis, which has potentially provided prognostic values (17–19). However, The research results on the prognostic significance of TILs and TAMs in tumors are inconsistent. Choo’s study have shown that a high proportion of CD8+ TILs suggests that tumour patients have a longer survival time (20). Zurlo’s research shows that GC patients with low CD4+/CD8+ ratios have better prognosis (21). On the contrary, another study by Jin et al. suggests that high-density TILs may indicate worse prognosis or two-way regulatory effect (21–23). Ren’s study shows that TILs does not affect the prognosis of GC patients (23). Similarly, studies on the prognostic significance of TAMs for GC patients also varied. Some studies have shown that TAMs can promote tumours progression, during the process of interactions with cancer cells, TAMs may undergo a phenotypic change, such as M1 macrophages transforming into M2 macrophages; indicating a worse prognosis for GC (24, 25). Whereas, other studies have suggested that TAMs infiltration suggests a better prognosis for GC (26). Due to different research methods, various subtypes and standards, the existing research makes it challenging to intricately explore the specific value of TIICs including TILs and TAMs.Conflicting results have led to disagreement on the type and association of these biomarkers to be used to investigate the GC-related immune condition for survival prediction (27–29). This debated topic suggests that simply analysing stratification based on TILs or TAMs may not be sufficient. The combination of TILs and TAMs distribution type would help establish a new model to. accurate prognostic prediction in GC in the future.Recently, immune checkpoints have been identified as negative regulators, limiting the effectiveness of anticancer responses and allowing for immune escape (23, 30, 31). In particular, PD-L1 expression on the surface of GC cells has been linked to a poor prognosis, larger tumors, and lymph node metastasis (32, 33). Recent research has focused on PD-L1 expression on the tumor cells and TIICs as the predictors of survival for GC patients (25, 34, 35). However, limited information is available on the relationship between PD-L1 expression and TIICs as well as its impact on the prognosis of GC patients. Furthermore, there is limited knowledge on the prognostic implications of the routine clinicopathological features when integrating PD-L1 expression and TIICs.

This retrospective study aims to evaluate the prognostic impact of PD-L1 expression and CD4+/CD8+ TILs as well as CD163+ TAMs, and to develop a prognostic model incorporating the expression of PD-L1 and related TIICs for survival prediction in GC patients.

## Materials and methods

This monocentric study was performed at Jiangnan University Medical Center, which was approved by the Institutional Ethics Review Boards. Informed consent was waived due to the retrospective nature.

### Patients data

**Figure 1:**
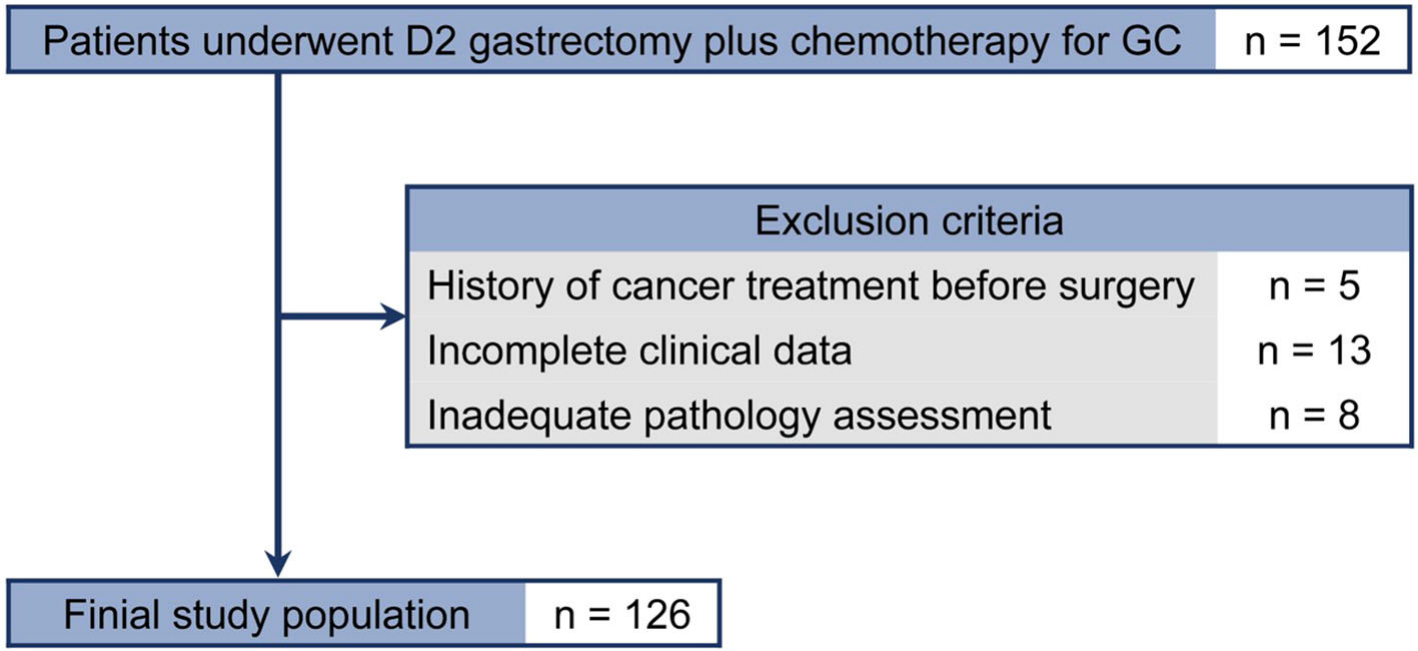
Flowchart of the present study. GC, gastric cancer.

Data from patients with pathologically identified GC who underwent D2 gastrectomy plus chemotherapy between January 2016 and May 2018 were reviewed(n=152). Exclusion criteria were: (1) history of cancer treatment before surgery(n=5); (2) incomplete clinicopathologic characteristics or no follow-up data(n=13); and (3) pathology slides not available for assessing PD-L1 expression and TIICs(n=8). Finally, 126 patients were included in the analysis (**Figure 1**).

Baseline information for each patient with GC, including age, gender, tumor location, tumor size, differentiation, Lauren classification, Her-2, Epstein-Barr virus, microsatellite instability, lymph node metastasis, vascular metastasis, pathological TNM stage after surgery, and follow-up data, were documented. 126 of the enrolled patients were treated with postoperative chemotherapy, 50 (39.7%) GC patients receiving the XELOX (capecitabine–oxaliplatin) regimen, and postoperative adjuvant chemotherapy was given 8 times in a cycle of 21 days, 76 (60.3%) GC patients receiving the FOLFOX (fluorouracil–folinic acid–oxaliplatin) regimen.and postoperative adjuvant chemotherapy was given 6 times in a cycle of14 days. 26 GC patients with stage III received neoadjuvant chemotherapy(NAC), the drugs mainly include fluorouracil and cisplatin, capecitabine, docetaxel, oxaliplatin. The enrolled patients were not received immunotherapy or targeted therapy.

Post-treatment follow-up assessments were conducted according to the institutional protocol, with carcinoembryonic antigen examined every 3 months. Abdominal/pelvic computer tomography scans were performed every 6 months, and gastroscopy was required annually. All patients were followed up every 3 months in the first 2 years, every 6 months in the 3–5 years, and every 12 months thereafter. DFS time was calculated from the date of surgery to the date of the first relapse or death. OS was defined as the interval from the date of surgery to the date of patient death or last follow-up. Follow-up ended in April 2023. The median observation period was 54.0 (95% CI, 47.9–60.1) months.

### Immunohistochemical assay

The selected paraffin blocks were cut into 4-μm-thick sections using a standard slicing machine. Immunohistochemical staining was performed using the Envision method, following the instructions of the kit (Maixin Biotechnology, Fuzhou, China;PD-L1 SP263,VENTANA,Roche,China). The primary antibody was diluted by 1:100 times. Phosphate-buffered saline was used as a blank control, PD-L1 was used with tonsil tissue and human placental tissue as positive controls, CD4, CD8 and CD163 were used with positive slices as positive controls. The slides were independently evaluated by two gastrointestinal pathologists blinded to clinicopathologic data. Discrepancies were re-evaluated, and a consensus decision was made.

**Figure 2:**
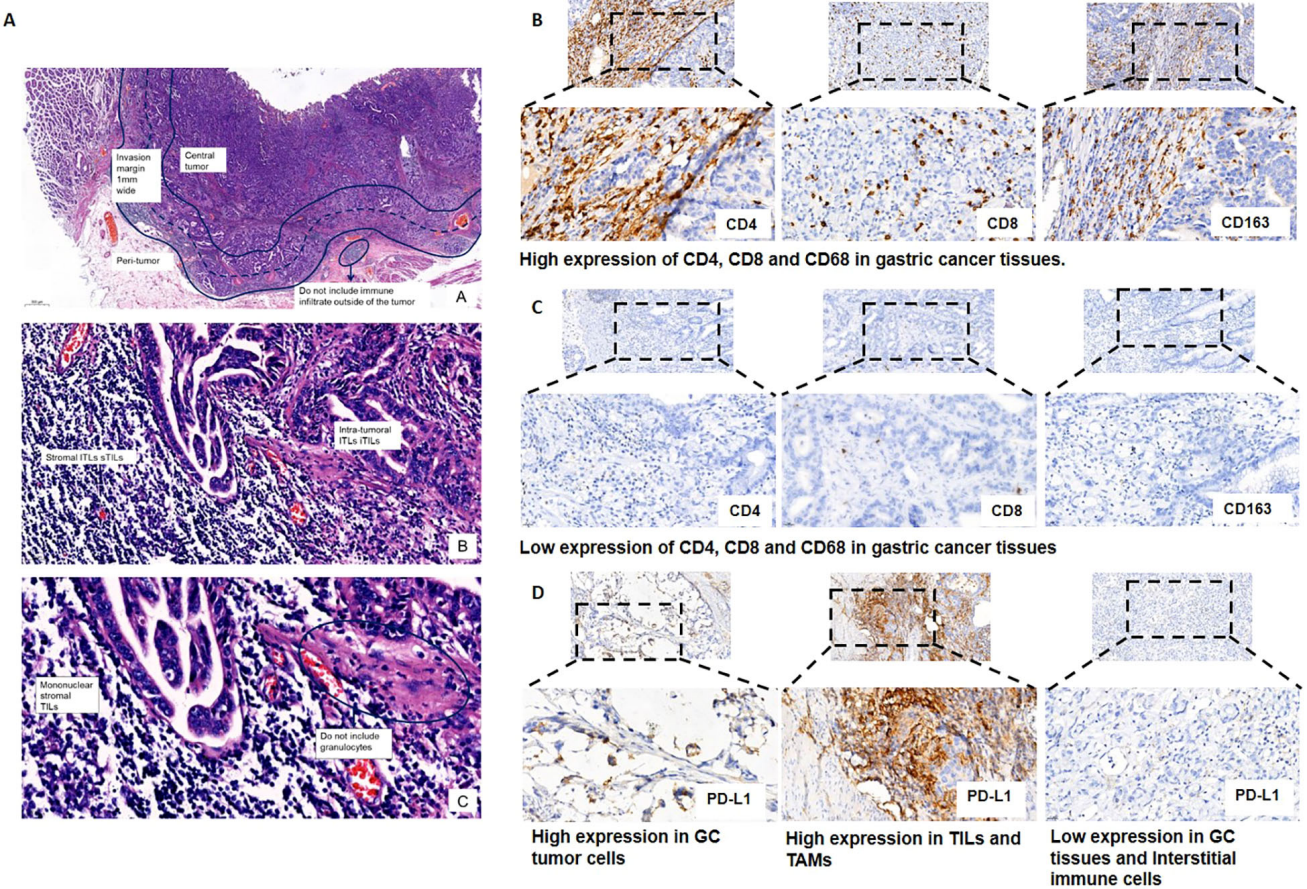
Representative images of the expression of tumor-infiltrating immune cells (i.e., CD4+ and CD8+ T lymphocytes and CD163+ macrophages) and programmed death ligand 1 (PD-L1). **(A)** Standardized approach for TILs and TAMs evaluation in gastric cancer tissues. Step 1: Scan at low magnification and select tumor area. The“invasive margin” (IM) is defined as the region centered on the border separating the host tissue from the malignant nests, with an extent of 1mm. “Central tumor” (CT) corresponds to all the tissue inside the IM, and “peritumor” (PT) to tissue outside of the IM. Please see this image in color online; Step 2: Define stromal and intra-tumoral areas; Step 3: Determine type of inflammatory infiltrate and assess the percentage TILs and TAMs. **(B)** High expression of CD4+ and CD8+ T lymphocytes and CD163+ macrophages in gastric cancer tissues. Staining was localized predominantly in the cytomembrane. **(C)** Low expression of CD4+ and CD8+ T lymphocytes and CD163+ macrophages in gastric cancer tissues. **(D)** Representative images of PD-L1 expression on tumor cells and tumor-infiltrating immune cells.


Staining of CD4, CD8 and CD163 in immune cells was estimated for TILs and TAMs on the marginal area of the tumor, including the epithelium and intratumor stroma by optical microscope (BX51, Olympus, Tokyo, Japan). Five noncontiguous areas, including the densest immune cells, were selected to ensure that the samples were representative and to increase homogeneity. The percentages of immune cells in the five fields were combined and then averaged to calculate the mean value for one 200× microscopic field. Mean values (CD4 + 22%, CD8 + 10%, CD163 + 4%) were utilized as cutoff values to categorize the CD4, CD8, CD163 expression levels for TILs and TAMs as “high” or “low”. (**Figures 2A–D**). PD-L1 positive expression shows intact or partial cytomembrane coloration of tumor cells, and cytoplasmic/membrane coloration of immune cells in the carcinoma stromal. To be considered adequate for evaluation, at least 100 viable tumor cells must be present in the PD-L1 stained slide. The Combined Positive Score (CPS) was used to evaluate PD-L1 expression, which is calculated by summing the number of PD-L1 stained cells (tumor cells, lymphocytes, macrophages) divided by the total number of viable tumor cells, multiplied by 100, as shown in the formula below.

$$\begin{array}{c} CPS=\\ \frac{number\;of\;PD-L1\;stained\;cells\;\left(tumor\;cells,\;lymphocytes,\;macrophages\right)\times 100}{\;Total\;number\;of\;viable\;tumor\;cells} \end{array}$$

### Statistical analysis

The clinical and pathological characteristics were described using medians and ranges for continuous variables and counts and percentages for categorical variables. Statistical comparison of continuous variables was performed using the Mann-Whitney *U* test or *t* test, as appropriate. Comparison of categorical variables was performed using the chi-square statistic or the Fisher’s exact test. Spearman correlation analysis was used to analyze the correlation.

Survival data were used to construct a univariate Cox proportional hazards model. Covariates that were significant at a *P* value of less than 0.1 were included in the multivariate Cox proportional hazards model. Through backward stepwise selection with Akaike information criterion as the stopping rule, the nomograms for DFS or OS were formulated based on the multivariate Cox regression analysis results. Model performance, discrimination, and calibration were measured by Harrell’s concordance-index (C-index), the time-dependent area under the receiver operating characteristic curve (tdAUC), and a calibration curve, respectively.10-fold cross-validation was used to validate the prediction models. The corrected C-indices were reported as measures of the performance of the models. With the effect of a variable with the highest coefficient (absolute value) being assigned 100 points, each coefficient in the multivariable regression obtained a point value proportionally. The summation of all point values was the total point of the nomogram. The minimum *P*-value approach was used to find the optimal number of prognostically distinct subgroups and corresponding cutoff point(s) for the total point of the nomogram. Cumulative DFS and OS rates were calculated using the Kaplan-Meier method, and differences between the curves were evaluated using the log-rank test.

All statistical analyses were performed using R version 4.1.1. *P*-values less than 0.05 were considered statistically significant.

## Results

### Patient characteristics

In this study cohort of 126 patients with GC, 70 (55.6%) were male. The median (interquartile range) age at diagnosis was 67.0 (55.0–74.5) years. Median DFS and OS were 36.0 (95% CI, 25.1–46.9) months and 48.0 (95% CI, 37.3–58.7) months, respectively. The clinicopathological characteristics of the patients are presented in **Table 1**.

**Table 1:** Patient characteristics according to the expression of PD-L1.

Characteristics	Subgroup	Total (n=126)	High PD-L1 expression (n=61)	Low PD-L1 expression (n=65)	*P* value
Gender	Male	70 (55.6%)	34 (55.7%)	36 (55.4%)	0.968
	Female	56 (44.4%)	27 (44.3%)	29 (44.6%)	
Age (years)	<70	68 (54.0%)	37 (60.7%)	31 (47.7%)	0.145
	≥70	58 (46.0%)	24 (39.3%)	34 (52.3%)	
Location	Cardia	34 (27.0%)	12 (19.7%)	22 (33.8%)	0.154
	Body	37 (29.4%)	18 (29.5%)	19 (29.2%)	
	Antrum	55 (43.6%)	31 (50.8%)	24 (37.0%)	
Lauren classification	Intestinal	68 (54.0%)	26 (42.6%)	42 (64.6%)	0.013
	Diffuse	58 (46.0%)	35 (57.4%)	23 (35.4%)	
Size	<4cm	65 (51.6%)	27 (44.3%)	38 (58.5%)	0.111
	≥4cm	61 (48.4%)	34 (55.7%)	27 (41.5%)	
Her-2	Negative	109 (86.5%)	50 (82.0%)	59 (90.8%)	0.148
	Positive	17 (13.5%)	11 (18.0%)	6 (9.2%)	
Epstein-Barr virus	Negative	114 (90.5%)	51 (83.6%)	63 (96.9%)	0.011
	Positive	12 (9.5%)	10 (16.4%)	2 (3.1%)	
Microsatellite instability	MSI-H	24 (19.0%)	17 (27.9%)	7 (10.8%)	0.002
	MSS	102 (81.0%)	44 (72.1%)	58 (89.2%)	
Lymph node metastasis	Negative	82 (65.1%)	30 (49.2%)	52 (80.0%)	<0.001
	Positive	44 (34.9%)	31 (50.8%)	13 (20.0%)	
Vascular metastasis	Negative	92 (73.0%)	31 (50.8%)	61 (93.8%)	<0.001
	Positive	34 (27.0%)	30 (49.2%)	4 (6.2%)	
TNM stage	I-II	86 (68.3%)	32 (52.5%)	54 (83.1%)	<0.001
	III	40 (31.7%)	29 (47.5%)	11 (16.9%)	
CD4	High	65 (51.6%)	39 (63.9%)	25 (38.5%)	0.004
	Low	61 (48.4%)	22 (36.1%)	40 (61.5%)	
CD8	High	24 (19.0%)	4 (6.56%)	20 (30.8%)	<0.001
	Low	102 (81.0%)	57 (93.44%)	45 (69.2%)	
CD163	High	51 (40.5%)	33 (54.1%)	17 (26.2%)	0.001
	Low	75 (59.5%)	28 (45.9%)	48 (73.8%)	

PD-L1, programmed death ligand 1; MSI-H, microsatellite instability-high; MSS, microsatellite stability; TNM, tumor–node–metastasis.

### Immunohistochemistry for TIICs and PD−L1 expression in GC tissues

In the study cohort of 126 GC patients, 65 (51.6%) had high expression of CD4+ TILs, 24 (19.0%) had high expression of CD8+TILs, and 51 (40.5%) had high expression of CD163+ TAMs (**Table 1**).

Among 126 enrolled patients, a high PD-L1 expression (CPS ≥ 5) was detected in 61 patients (48.4%), while a low PD-L1 expression (CPS < 5) was detected in 65 patients (51.6%). Correlating the PD-L1 expression with the clinicopathological characteristics of the study cohort, we found that Lauren type, Epstein-Barr virus, microsatellite instability, lymph node metastasis, vascular metastasis,TNM stage, CD4+/CD8+ TILs and CD163+ TAMs were significantly associated with PD-L1 expression. In contrast, none of the other characteristics showed any significant correlation (**Table 1**).

### Relationship between PD-L1 expression levels and CD4 +/CD8+TILs, CD163+ TAMs density in GC patients

**Figure 3:**
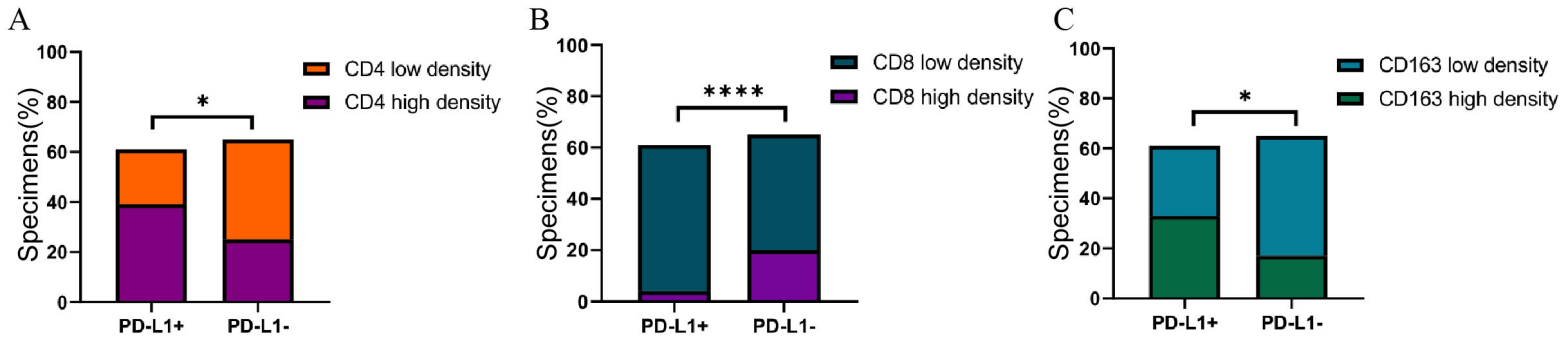
**(A)** Association of PD-L1with CD4 expression in GC specimens. **(B)** Association of PD-L1with CD8 expression in GC specimens. **(C)** Association of PD-L1with CD163 expression in GC specimens. Correlation was evaluated by Spearman test. *P<0.05, ****P<0.0001.

PD-L1 expression levels was negatively correlated with CD8+TILs density (*P*<0.0001), and positively correlated with CD4+ TILs and CD163+ TAMs density (*P*<0.05) (**Figure 3**).

### Prognostic variables and prediction models for DFS and OS

All clinicopathological variables and the expression of TIICs and PD−L1 in **Table 1** were included in Cox regression analyses. The results of univariable analyses were showed in **Table 2**. After univariable and multivariable analyses, with results reported as HR with 95% CI, high PD-L1 expression (2.17 [1.37–3.43]), low CD8+TILs density (0.35[0.15–0.81]), high CD163+ TAMs density (1.84[1.17–2.89]), MSI-H(MSI-H vs. MSS, 0.41 [0.20–0.83]) and TNM stage (stage III vs stage I+II,1.37[1.06–2.23]) were independently associated with DFS (**Table 3**). Additionally, high PD-L1 expression (2.64 [1.61–4.34]), high CD4+ TILs density(1.98 [1.21–3.24]), low CD8+ TILs density(0.23 [0.07–0.73]), high CD163+ TAMs density (2.31 [1.43–3.74]), MSI-H(MSI-H vs. MSS, 0.26 [0.12–0.60]) and TNM stage (stage III vs. stage I+II, 1.61 [1.01–2.56]) were independently associated with OS (**Table 3**).

**Table 2:** Univariable Cox regression analyses for predicting DFS and OS.

Variables	Subgroup	DFS	*P* value	OS	*P* value
		HR (95% CI)		HR (95% CI)	
Gender	Male	Ref		Ref	
	Female	0.82 (0.53–1.30)	0.392	0.85 (0.53–1.30)	0.485
Age(years)	<70	Ref		Ref	
	≥70	0.81 (0.52–1.30)	0.344	0.83 (0.52–1.30)	0.436
Location	Antrum	Ref		Ref	
	Cardia	1.25 (0.75–2.09)	0.392	1.19 (0.70–2.02)	0.529
	Body	0.64 (0.37–1.10)	0.107	0.58 (0.33–1.04)	0.068
Lauren classification	Intestinal	Ref		Ref	
	Diffuse	0.84 (0.54–1.30)	0.423	0.89 (0.56–1.40)	0.614
Size	<4cm	Ref		Ref	
	≥4cm	0.97 (0.62–1.50)	0.878	0.96 (0.61–1.50)	0.849
Her-2	Negative	Ref		Ref	
	Positive	1.05 (0.57–1.90)	0.871	1.17 (0.63–2.20)	0.615
Epstein-Barr virus	Negative	Ref		Ref	
	Positive	0.33 (0.10–1.00)	0.059	0.24 (0.06–0.97)	0.045
Microsatellite instability	MSI-H	Ref		Ref	
	MSS	0.52 (0.26–1.00)	0.067	0.43 (0.20–0.93)	0.033
Lymph node metastasis	Negative	Ref		Ref	
	Positive	1.31 (0.84–2.00)	0.235	1.41 (0.89–2.20)	0.143
Vascular metastasis	Negative	Ref		Ref	
	Positive	1.69 (1.10–2.70)	0.027	1.75 (1.10–2.80)	0.021
TNM stage	I-II	Ref		Ref	
	III	1.53 (0.98–2.40)	0.064	1.77 (1.10–2.80)	0.015
PD-L1	Low	Ref		Ref	
	High	1.83 (1.20–2.90)	0.008	1.96 (1.20–3.10)	0.005
CD4	Low	Ref		Ref	
	High	1.55 (0.99–2.40)	0.056	1.96 (1.20–3.20)	0.006
CD8	Low	Ref		Ref	
	High	0.29 (0.13–0.68)	0.004	0.15 (0.05–0.47)	0.001
CD163	Low	Ref		Ref	
	High	1.95 (1.30–3.00)	0.003	2.32 (1.50–3.70)	<0.001

DFS, disease-free survival; OS, overall survival; HR, hazard ratio; CI, confidence interval; MSI-H, microsatellite instability-high; MSS, microsatellite stability; TNM, tumor–node–metastasis; PD-L1, programmed death ligand 1.

**Table 3:** Final model of the multivariate COX regression analysis for predicting DFS and OS.

Variables	Coefficient	HR	95% CI	*P* value
Prediction model for DFS				
High PD-L1 expression	0.775	2.17	1.37–3.43	0.003
High CD8 level	-1.059	0.35	0.15–0.81	0.017
High CD163 level	0.608	1.84	1.17–2.89	0.016
Microsatellite instability-high	-0.893	0.41	0.20–0.83	0.020
TNM stage (III vs I+II)	0.426	1.37	1.06–2.23	0.046
Prediction model for OS				
High PD-L1 expression	0.971	2.64	1.61–4.34	<0.001
High CD4 level	0.683	1.98	1.21–3.24	0.006
High CD8 level	-1.488	0.23	0.07–0.73	0.013
High CD163 level	0.837	2.31	1.43–3.74	<0.001
Microsatellite instability-high	-1.331	0.26	0.12–0.60	0.001
TNM stage (III vs I+II)	0.476	1.61	1.01–2.56	0.044

DFS, disease-free survival; OS, overall survival; HR, hazard ratio; CI, confidence interval; PD-L1, programmed death ligand 1; TNM, tumor–node–metastasis.

**Figure 4:**
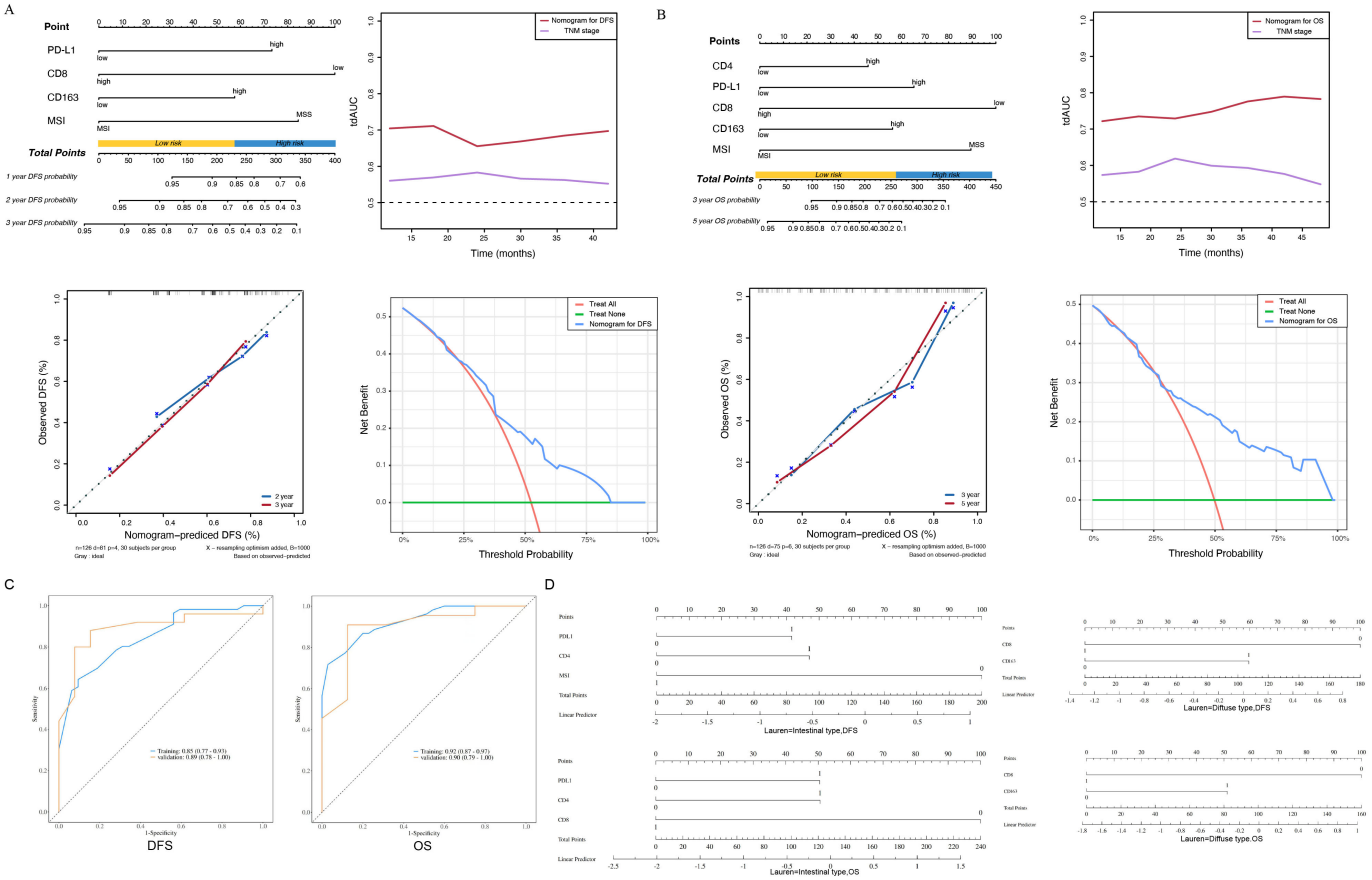
**(A)** The nomogram, the time-dependent area under receiver operating characteristic curve, the calibration curve and the decision curve of the prognostic model for disease-free survival (DFS). PD-L1, programmed death ligand 1; MSI-H, microsatellite instability-high; MSS, microsatellite stability. **(B)** The nomogram, the time-dependent area under receiver operating characteristic curve, the calibration curve and the decision curve of the prognostic model for overall survival (OS). TNM, tumor–node–metastasis; PD-L1, programmed death ligand 1; MSI-H, microsatellite instability-high; MSS, microsatellite stability. **(C)** The ROC curve of the prognostic model for disease-free survival (DFS), The ROC curve of the prognostic model for overall survival (OS). **(D)** Intestinal type of subgroup analysis based on Lauren classification for disease-free survival (DFS) and overall survival (OS), Diffuse type of subgroup analysis based on Lauren classification for disease-free survival (DFS) and overall survival (OS).

Four predictive factors (i.e., PD-L1, CD8+ TILs, CD163+ TAMs and MSI-H) were adopted in the final DFS prediction model for the construction of the nomogram (**Table 3**). The established nomogram for DFS yielded a C-index of 0.687 (95% CI, 0.618–0.760), a corrected C-index of 0.679 by 10-fold cross-validation. Comparisons of the performance and discrimination between the model and TNM stage showed the tdAUC of the nomogram for DFS is consistently higher than the TNM stage over time. The 2- and 3-year calibration curves showed the correction curve fits well with the standard curve, with its decision curve showed that when the threshold range is between 20% and 80%, the nomogram has superior diagnostic value in predicting prognosis (**Figure 4A**).

Five predictive factors (i.e., PD-L1, CD4+ TILs, CD8+ TILs, CD163+ TAMs, MSI-H) were adopted in the OS prediction model for the nomogram construction (**Table 3**). The established nomogram for OS yielded a C-index of 0.749 (95% CI, 0.685–0.810), and a corrected C-index of 0.755 by 10-fold cross-validation. Comparisons of the performance and discrimination between the model and TNM stage showed that the tdAUC of the nomogram for OS higher than the TNM stage. The 3- and 5-year calibration curves showed the correction curve fits well with the standard curve, with its decision curve showed that when the threshold range is between 30% and 90%, the nomogram has superior diagnostic value in predicting prognosis (**Figure 4B**).

We conducted ROC curve analysis on the DFS and OS groups of the nomograms. The AUC value of the nomograms model based on the above four predictive factors for DFS prediction is 0.85, and the AUC value of the nomograms model based on the above five predictive factors for OS is 0.90, indicating effective prediction performance (**Figure 4C**). Based on the above research, we added subgroup analysis based on Lauren classification. In the subgroup analysis for predicting the prognosis of intestinal type gastric cancer, it was shown that PDL1, CD4, MSI-H were closely related to gastric cancer DFS, while PDL1, CD4+TILs, CD8+TILs were closely related to gastric cancer OS. Among them, the expression of MSI-H and CD8+TILs suggested a good prognosis, while the expression of PDL1 and CD4+TILs suggested a poor prognosis. In the subgroup analysis of prognosis prediction for diffuse gastric cancer, CD8+TILs, CD163+TAMs are closely related to gastric cancer DFS and OS. Among them, the expression of CD8+TILs suggested a good prognosis, while the expression of CD163+TAMs suggested a poor prognosis. There are some differences between subgroup analysis and overall analysis, which may be related to the different immune microenvironments of various subtypes of gastric cancer (**Figure 4D**).

**Figure 5:**
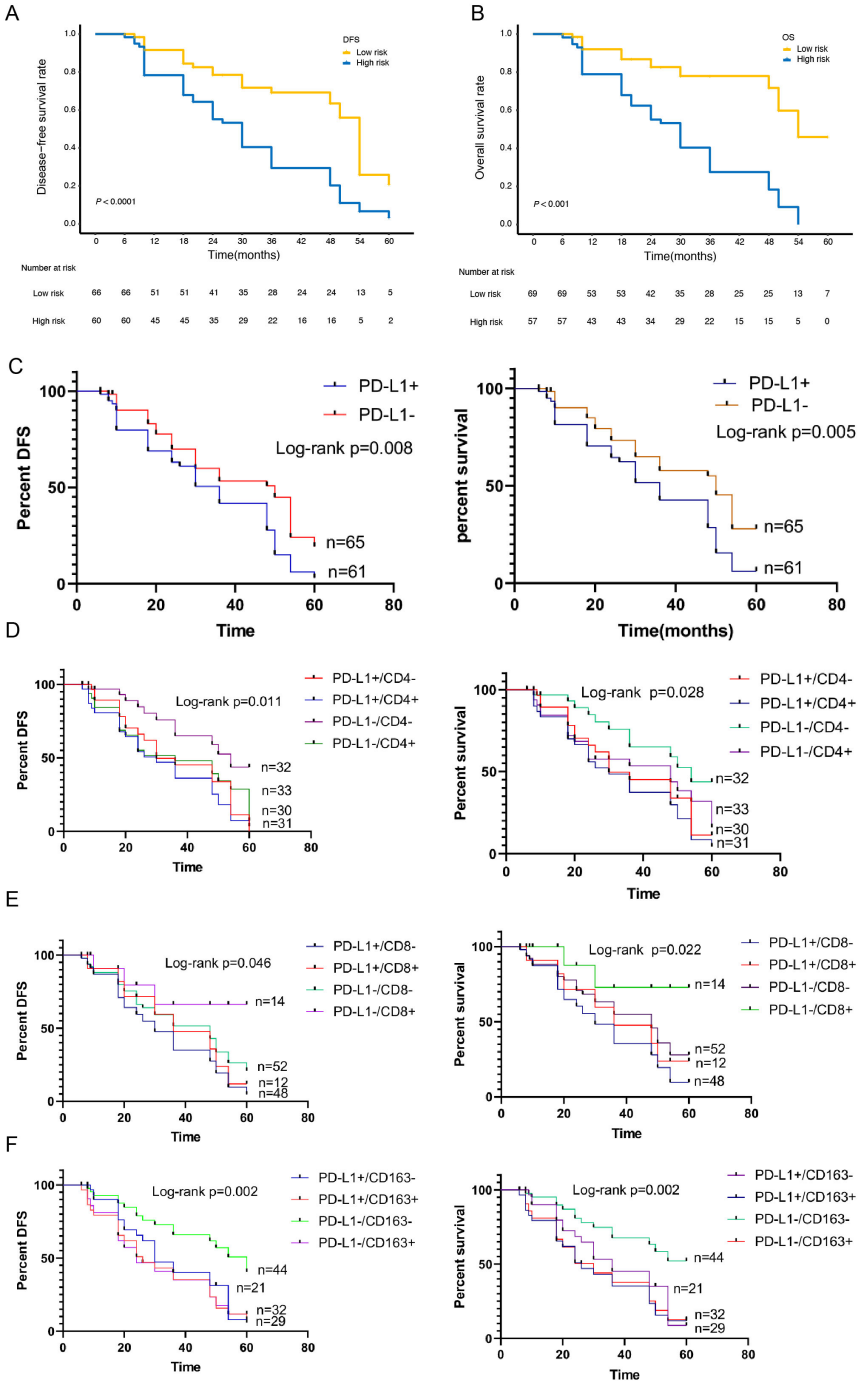
Kaplan-Meier survival curves of the Subgroup analysis according to the expression of PD-L1, CD4, CD8 and CD163. **(A)**Kaplan-Meier survival curves of the 2 strata patients which were stratified by the models for disease-free survival and **(B)** overall survival. **(C)** Kaplan-Meier survival curves of the Subgroup analysis according to the expression of PD-L1 for disease-free survival and overall survival. **(D)** Kaplan-Meier survival curves of the Subgroup analysis according to the expression of PD-L1/CD4 for disease-free survival and overall survival. **(E)** Kaplan-Meier survival curves of the Subgroup analysis according to the expression of PD-L1/CD8 for disease-free survival and overall survival. **(F)** Kaplan-Meier survival curves of the Subgroup analysis according to the expression of PD-L1/CD163 for disease-free survival and overall survival.

Patients were stratified into two prognostically distinct groups for DFS and OS, respectively, according to the cutoff points of the nomogram-predicted score identified by the minimum *P*-value approach. According to the cutoff points of the DFS nomogram-predicted score, patients were divided into two strata: a low-risk group, with a predicted score of no more than 230, and a high-risk group, with a predicted score of more than 230. Having the low-risk group as a reference, the HR for the high-risk group was 2.717 (95% CI, 1.681–4.392; *P*<0.001; **Figure 5A**). Similarly, according to the cutoff points of the OS nomogram-predicted score, patients were divided into two strata: a low-risk group, with a predicted score no more than 270, and a high-risk group, with a predicted score of more than 270. Having the low-risk group as a reference, the HR for the high-risk group was 4.620 (95% CI, 2.729–7.822; *P*<0.001; **Figure 5B**).

### Subgroup survival analysis of the relationship between PD-L1 and TIICs

To further verify the related predictive effect, we performed a subgroup survival analysis of the relationship between PD-L1 and TIICs. The results showed that the OS and DFS of PD-L1 positive group was significantly lower than that of negative group (*χ* ^2^ = 8.030, *P*=0.005; *χ* ^2^ = 7.108, *P*=0.008) (**Figure 5C**).

According to the expression of PD-L1 and CD4+TILs, it was divided into PD-L1+/CD4+ group, PD-L1+CD4- group, PD-L1-CD4+ group and PD-L1-CD4- group. Survival analysis showed that the difference of OS and DFS between the four groups was statistically significant (*χ* ^2^ = 9.135, *P*=0.028; *χ* ^2^ = 11.09, *P*=0.011). The OS and DFS was the highest in PD-L1/-CD4- group and the lowest in PD-L1+/CD4+ group (**Figure 5D**). According to the expression of PD-L1 and CD8+TILs, it was divided into PD-L1+/CD8+ group, PD-L1+/CD8- group, PD-L1-/CD8+ group and PD-L1-/CD8- group. Survival analysis showed that the OS and DFS was the highest in PD-L1-/CD8+ group and the lowest in PD-L1+/CD8- group. There was significant difference in OS and DFS between the four groups (*χ* ^2^ = 9.603, *P*=0.022; *χ* ^2^ = 7.986, *P*=0.046)(**Figure 5E**).According to the expression of PD-L1 and CD163+TAMs, it was divided into PD-L1+/CD163+ group, PD-L1+/CD163- group, PD-L1-/CD163+ group and PD-L1-/CD163- group. Survival analysis showed that the OS and DFS in PD-L1-/CD163- group was the highest, and that in PD-L1+/CD163+ group was the lowest. There was a statistically significant difference between the four groups (*χ* ^2^ = 15.21, *P*=0.002; *χ* ^2^ = 14.46, *P*=0.002) (**Figure 5F**).

### Internal validation of prediction models for DFS and OS

**Figure 6:**
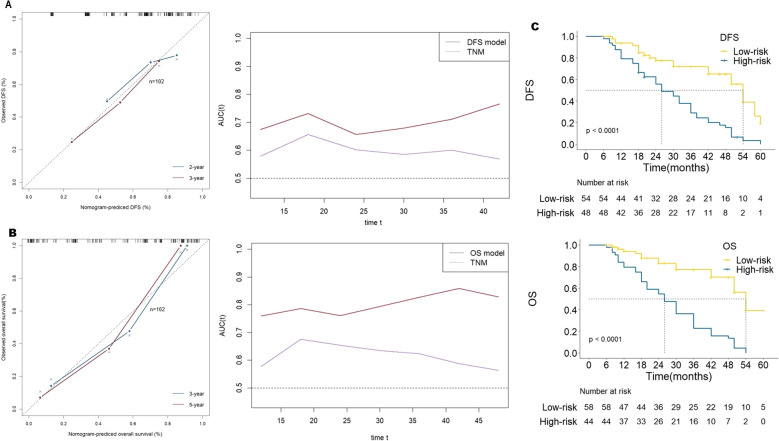
**(A)** the calibration curve of the prognostic model for disease-free survival (DFS) and the time-dependent area under receiver operating characteristic curve by internal validation of prediction models. **(B)** the calibration curve of the prognostic model for overall survival (OS) and the time-dependent area under receiver operating characteristic curve by internal validation of prediction models. **(C)** Kaplan-Meier survival curves of the 2 strata patients which were stratified by the models for disease-free survival and overall survival by internal validation of prediction models.

In order to confirm the predictive value of the nomogram based on the above multivariates, we selected 102 patients with GC for internal verification, and came to the same inclusion. Data from patients with pathologically identified GC who underwent D2 gastrectomy plus chemotherapy between January 2018 and May 2021 were reviewed. Four predictive factors (i.e., PD-L1, CD8+ TILs, CD163+ TAMs and MSI-H) were adopted in the DFS prediction. The established nomogram for DFS yielded a C-index of 0.695(95% CI,0.618-0.770), a corrected C-index of 0.641 by 10-fold cross-validation. The tdAUC of the nomogram for DFS is higher than the TNM stage (**Figure 6A**).

Five predictive factors (i.e., PD-L1, CD4+TILs, CD8+TILs, CD163+TAMs, MSI-H) were adopted in the OS prediction model. The established nomogram for OS yielded a C-index of 0.774(95% CI, 0.709-0.840), and a corrected C-index of 0.678 by 10-fold cross-validation. The tdAUC of the nomogram for OS consistently higher than the TNM stage (**Figure 6B**).

According to the cutoff points of DFS predicted score, patients were divided into low-risk group and high-risk group. The HR for the high-risk group compared with the low-risk group was 3.413 (95% CI, 2.01-5.79), P< 0.001. Similarly, according to the cutoff points of OS predicted score, the HR for the high-risk group compared with the low-risk group was 4.846 (95% CI, 2.74-8.57), P< 0.001 (**Figure 6C**).

## Discussion

PD-L1 and TIICs have been demonstrated as potential markers for predicting survival outcomes in GC, however, the performance of integrating these markers for survival prediction remains poorly understood (32, 33, 36). In this study, we proposed prediction models for DFS and OS in GC, incorporating these markers and clinical features, with a C-index of 0.679 and 0.755 after 10-fold cross-validation, respectively. The results of the above experiments were confirmed by internal validation. These results suggest that the nomograms integrating the expression of PD-L1 and TIICs could provide an accurate prediction of prognosis in GC.

Considering the large variations in the clinical outcomes of GC, the traditional TNM staging, which was based mainly on anatomical information, was suboptimal in prognostic prediction (8–10). Therefore, it was necessary to develop new methods based on important clinicopathologic variables other than traditional TNM staging to improve prognostic prediction for GC (11, 12). As both disease progression and survival information are essential for personalized prognosis, DFS and OS were used as the endpoints in the present study. The results showed that the expression of PD-L1 and TIICs were valuble prognostic factors. Furthermore, the proposed models integrating the bioimmunological characteristics of GC outperformed the TNM stage.

In recent years, research into immune markers has been at the forefront of GC studies (11, 19, 37). Several studies have demonstrated that CD4+ TILs and CD8+ TILs play different roles in predicting the prognosis of GC (38, 39). CD4+ TILs may be one of the main factors that have an immunosuppressive role, wheras, CD8+ TILs indicates a good prognostic marker. TAMs can be divided into M1 type and M2 type.M1 type TAMs expresses CD68 and promotes the inflammatory response, which usually has an anti-tumour effect. On the contrary, M2 type TAMs expresses CD163, have anti-inflammatory and pro-angiogenic effects, and they can maintain tissue dynamic homeostasis and fibrin production and inhibit antitumour responses similar to those of M1 macrophages, indicating a worse outcome (38). TAMs usually exhibit an M2-like phenotype and may have a strong immunoreactive function in the initial stages of cancer; in later stages, the microenvironment is enriched with growth factors and anti-inflammatory mediators such as IL-4, IL-10 and transforming growth factor-b(TGF-b), which induce macrophage polarisation, and the cells thus acquire an M2 phenotype with tumour-promoting functions Macrophages polarise towards the M1 phenotype in response to factors such as lipopolysaccharide (LPS), IFN-g, and TNF-a, which play a proinflammatory role and immune function. In contrast, genetic evidence suggests that TH2 cell-derived IL-4 and IL-13 may play a key role in the M2 polarisation of macrophages. It is reported that M2 type macrophages can induce PD-L1 expression through a variety of cytokines and signal pathways to promote GC cells to escape the killing of cytotoxic T cells (19, 40, 41). Therefore, incorporating various types of TILs and TAMs into the prognostic model of GC may have specific guiding significance for the clinical diagnosis and treatment of GC.

The present study showed that CD4+ TILsand CD163+ TAMs were negatively significantly correlated with OS in patients with GC, as previously reported (19, 26, 29). However, high-level expression of CD8+ TILs was found to be positively correlated with DFS and OS, indicating that CD8+ TILs may be as an index of a patient’s better immune response (20, 29, 42).

PD-L1 has received considerable attention due to its biological and prognostic implications (31–33). Our results suggest that PD-L1 positivity expression predict a worse prognosis, which is consistent with the previous studied. Although there is still controversy over the relationship between PD-L1 expression and prognosis in GC, most studies believe that PD-L1 is a negative prognostic predictor. A recent meta-analysis covering 15 eligible studies with 3291 patients showed that the expression level of PD-L1 negatively correlated with the OS of GC. In addition to, subgroup analysis showed GC patients with deeper tumor infiltration, positive lymph-node metastasis, positive venous invasion, EBV+, MSI-H are more likely to expression PD-L1, suggesting that GC patients specifically with EBV+ and MSI-H may be prime candidates for PD-1 directed therapy. These findings support PD-L1 can serve as a valuable marker of prognostic prediction and immunotherapy for GC (13, 23, 43). According to the reports in literature, CPS ≥ 5 of PD-L1 expression contributed to the evaluation of immunotherapy in GC (44, 45). In univariate and multivariate analysis, Our data suggest that PD-L1 is significantly correlated with worse DFS and OS (both *P*< 0.05), and is likewise significantly associated with Lauren type, Epstein-Barr virus, microsatellite instability, lymph node metastasis, vascular metastasis and TNM stage, which is in line with the reported literature (46). However, the expression of PD-L1 alone may not be sufficient to predict survival for GC accurately. In this study, it is indicated that TIICs were significantly correlated with PD-L1 expression in GC progression. The expression of PD-L1 was positively correlated with CD4+ TILs and CD163+ TAMs density, whereas negatively correlated with CD8+ TILs density.

The subgroup analysis showed that the prognosis of patients was better in PD-L1-/CD4- group, PD-L1-/CD8+ group and PD-L1-/CD163- group, while was worse in those with PD-L1+/CD4+ group, PD-L1+/CD8- group and PD-L1+/CD163+ group. It is indicated that CD8+ phenotype with effector T cell inflammation may be as an index of a patient’s better immune response state by reducing PD-L1 expression. On the contrary, the CD4+ TILsand CD163+ Tams promotes GC cells to escape the killing of cytotoxic T cells by inducing PD-L1 expression, suggesting a poor immune response state. It was noteworthy that microsatellite instability provided stably prognostic value in the proposed nomograms as reported (47).Taking together, the main shortcomings of traditional TNM staging are excessive reliance on anatomical information, lack of consideration at the molecular biological levels and tumor related immune microenvironment types, inability to reflect the dynamic changes and individual differences of tumors. Comparisons of the performance and discrimination between the model and TNM stage showed that the tdAUC of the nomogram for DFS was consistently higher than the TNM stage over time, indicating significant superiority in predicting prognosis compared to TNM staging. In addition, in the research field of immune microenvironment markers for GC, most previous studies mainly focused on analyzing the expression of PD-L1 or TILs and TAMs separately, without forming a predictive model. Yang’s study used multiple immunohistochemistry to detect the expression of PD-L1 and multiple TIICs, and constructed a multidimensional TIIC model to predict the response to immunotherapy, but there are also some shortcomings. Although the m-IHC method used is advantageous over traditional IHC, a consensus set of protein markers has not yet been defined, and therefore differences in marker selection will exist. Moreover, the high cost and complex data analysis limit the application of this technology (48). In our study, the proposed nomograms were based on PD-L1 expression and TIICs density as well as parameters that are routinely assessed during postoperative workup, providing convenient tools for assessing the risk of disease progression and survival to screen for TIICs subtypes with different prognosis and establishing individualized case management plans after resection. High-risk indications may prompt doctors to increase the frequency of patient visits and introduce immunotherapy. Therefore, the current nomograms could be used to predict survival and potentially guide decisions around immunotherapy.

This study has some limitations. The established nomogram for DFS and OS yielded the C-index was 0.687 and 0.749 respectively, which was located at a medium level, suggesting that it had an effective predictive function for the prognosis of GC and needed to be further optimized through more precise improvement strategies. Firstly, The immune microenvironment of GC exhibits high heterogeneity, with differences in immune cell composition, immune checkpoint expression, and immune suppression mechanisms among different subtypes, which may lead to some bias in the results of the analysis. it was a retrospective, single-center study, the results need to be validated by prospective, larger, multi-center trials to reduce the risk of overfitting. Additionally, the mechanism behind the predictive value of the nomograms is not well understood. we added subgroup analysis based on Lauren classification. There are some differences between subgroup analysis and overall analysis, which may be related to the different immune microenvironments of various subtypes of gastric cancer, and further subtype stratification analysis may provide more insight into the roles of these features in the progression of GC and aid in treatment decision-making. Another limitation was that the regulatory mechanism of TME is very complex, various immune cell components interact closely with cancer cells, and then interact with each other to promote tumour development, other biomarkers were not considered in this study that may improve the accuracy and predictive value of the nomograms. Therefore, the performance of the nomograms may be further enhanced by incorporating additional markers to establish a more accurate multidimensional evaluation system.

## Conclusions

In conclusion, this study shows that the prediction models combining the expression of PD-L1 and CD4+/CD8+ TILs as well as CD163+ TAMs can accurately distinguish GC patients with substantially different DFS and OS. The developed nomograms may help to stratify prognosis, make decisions about personalized treatment, and plan follow-up schedules.

## Data Availability

The original contributions presented in the study are included in the article/supplementary material. Further inquiries can be directed to the corresponding authors.
